# Effect of Wet and Dry Finishing and Polishing Technique on Microhardness and Flexural Strength of Nanocomposite Resins

**DOI:** 10.1155/2023/2182094

**Published:** 2023-02-16

**Authors:** Amir Ghasemi, Anoosh Mohammadzadeh, Mehdi Molaei, Seyedeh Mahsa Sheikh-Al-Eslamian, Mandana Karimi

**Affiliations:** ^1^Department of Restorative Dentistry, Dental School, Shahid Beheshti University of Medical Sciences, Tehran, Iran; ^2^Dental Research Center, Research Institute of Dental Sciences, Shahid Beheshti University of Medical Science, Tehran, Iran; ^3^Preventive Dentistry Research Center, Research Institute of Dental Sciences, Dental School, Shahid Beheshti University of Medical Sciences, Tehran, Iran; ^4^Department of Restorative Dentistry, Dental School, Tehran University of Medical Sciences, Tehran, Iran

## Abstract

**Objectives:**

This in vitro study was aimed to assess the effect of wet and dry finishing and polishing techniques on the flexural strength and microhardness of different commercial nanoparticle contained composite resins.

**Methods and Materials:**

The samples were made of Z250 (microhybrid), Z350 XT (nanofilled), and Z550 (nanohybrid) resin composites. Each group was subdivided into 2 subgroups according to polishing protocols. Subgroup 1 for each composite underwent wet polishing, and subgroup 2 was subject to dry polishing technique. Flexural strength and microhardness of the samples were measured at two different times of polishing (*T*_0_ and *T*_24_). The flexural strength test and microhardness test were measured by a 3-point bending test using a universal testing machine, and a Vickers machine, respectively. Data were analyzed by Kolmogorov–Smirnov, two-way ANOVA, and Tukey HSD tests.

**Results:**

ANOVA showed that the type of composite has a significant effect on flexural strength. Two-way ANOVA showed that, at *T*_0_, flexural strength of all composites in the dry technique was higher than in the wet technique (*p* = 0.019). At *T*_24_, Z350 XT had the lowest, and Z250 had the highest flexural strength in both techniques. The time and technique of polishing were also significantly effective on hardness. At *T*_0_, hardness was higher in the wet compared to the dry method (*p* = 0.008). Tukey test showed that, at *T*_24_, the hardness of Z350 XT was significantly higher than the other materials in both techniques.

**Conclusion:**

Immediate wet finishing and polishing presented lower flexural strength. Delayed dry/wet finishing and polishing significantly enhanced the hardness of the samples.

## 1. Introduction

Nowadays, not only patients but clinicians also prefer composite resins as a tooth-colored material because of their good aesthetic quality and physical and mechanical properties [1]. A large part of the weight and volume of composite resins consists of fillers [2]. Fillers are responsible for strengthening the resin matrix, creating translucency, and abating the shrinkage caused by polymerization. More filler particles facilitate the clinical application of composite resins. Also, the particle size of fillers has a distinct effect on the mechanical properties of particulate-polymer composites. Fillers with smaller particle sizes have increased particle surface area, which results in a high surface energy at the filler-matrix interface [2]. Therefore, the smaller the size of the filler particles, the more they can coalesce into the structure of the composite material [2]. Following progression in nanoparticle development, it has been postulated that nanocontained resin composites possess favorable mechanical properties. Adding nanoparticle fillers to the resin matrix of dental composites improves aesthetic, optical, mechanical properties, wear resistance, and gloss retention and reduces polymerization shrinkage [2].

The uneven or rough surface of a restoration due to improper finishing and polishing leads to staining, accumulation of plaque, recurrent carries, wear, and reduced longevity and durability of the restoration [3]. Thus, finishing and polishing procedures are required for dental restorations to meet the requisite aesthetic, function, and periodontal health properties of dental restorations [4, 5].

Different methods are available for finishing and polishing. The composition of the composite, the presence of air incorporated in the composite, and the instruments used are the most important factors which can influence the surface glossiness and smoothness [6].

The best time to finish and polish composite restorations is a controversial matter. Although composite manufacturers recommend that the procedure must be better carried out immediately after restoration, research shows that it must be postponed to prevent the side effects of heat generation including smearing the resin matrix and creating local hotspots before the final polymerization of the composite [7]. But the fact is that delayed finishing and polishing can also cause disadvantages such as lower microhardness. This may be related to the loss of surface properties after polymerization and the stress produced during the delayed polishing procedure. In this way, delayed polishing procedures can compromise the marginal sealing obtained with the hygroscopic expansion of the composite and adhesive system, resulting in an increase in microleakage due to the stresses and the heat generated by the procedures. However, in the immediate polishing technique, hygroscopic expansion after several days can compensate for the damage caused by the procedures and improve the marginal sealing [8].

Besides the time of polishing, the method of polishing can also be considered of great importance. Polishing can be carried out by dry or wet methods. The dry method can cause a better view and administration of the work area, but it generates a lot of heat, which can affect the restoration properties. In the wet technique, a water coolant is used to decrease the temperature in order to prevent the damages caused by heat [9].

There is no consensus on which condition provides the best surfaces for nanoparticle contained resin composites. The objective of the present study was to evaluate the effect of immediate or delayed dry and wet finishing/polishing on the surface hardness and flexural strength of composite resins.

## 2. Materials and Methods

In this *in vitro* study, 3 types of composite resins were investigated: microhybrid (Filtek Z250 3M, ESPE, USA), nanofilled (Filtek Z350XT 3M, ESPE, USA), and nanohybrid (Filtek Z550 3M, ESPE, USA). In Table 1, the materials, product names, manufacturers, and compositions are listed.

### 2.1. Specimens Preparation for Flexural Test

The samples for this test were prepared as per ISO 4049 standards. 20 samples were made from each material according to a similar previous study [5]. A stainless steel mold of 25 × 2 × 2 mm was used. The mold was placed on a glass slab. The composite was packed into the mold until it was full. Then, a transparent mylar strip was placed on the composite and a glass slab over the mylar strip. In order to eliminate the excess composite, the glass lam was pressed slightly. Then, using a halogen light cure device (Optilux 501, Kerr Manufacturing Inc, Orange, CA, USA), each specimen was cured in 3 areas with a light intensity of 600 mW/cm^2^ for 60 seconds according to the manufacturer's instructions. First, the central area and then the two sides were cured using an overlapping manner to make sure that all areas have been covered. Then, the specimens were taken out of the mold and their dimensions were measured using a digital caliper (Digimatic caliper, Mitutoyo Corp., Tokyo, Japan).

### 2.2. Specimens Preparation for Hardness Test

Twenty disk shape samples of each composite were made with a 6 mm diameter and 2 mm height. The method for preparing samples was similar to that of the flexural strength test except for mold shape. Curing was carried out using a halogen light cure device (Optilux 501, Kerr Manufacturing Inc, Orange, CA, USA) with an intensity of 600 mW/cm^2^ for 20 seconds according to the manufacturer's instructions. The tip of the device was in complete contact with the covering glass tip, and perpendicular to the center of the sample.

### 2.3. Finishing and Polishing Procedures

Then, samples of each composite were randomly divided into 4 groups as follows: group *W*t_0_: finished and polished immediately under wet technique, group *D*t_0:_ finished and polished immediately under dry technique, group W*t*_24_: finished and polished after 24 hours with wet technique, and group *Dt*_24_: finished and polished after 24 hours with dry technique.

All finishing and polishing procedures were performed by the same operator, who was blinded to the group allocation of samples. Finishing and polishing were carried out using OptiDisc Kit (KerrHawe SA, Switzerland). The disks were used in the order of medium, fine, and extrafine grit sizes by a low-speed (5000 rpm) hand piece (Ti-Max Electric hand piece; NSK, Tokyo, Japan).

For dry samples each specimen was polished for 15 seconds by each disk, using a rotating movement with slight constant pressure. For wet samples, the same procedure was carried out, but it was carried out using a waterjet with a flow rate of 20 cc/min as coolant. For dry groups, between changing the disks, the sample was cleaned with a soft tissue paper to remove the debris, while for the wet samples, they were washed with water for 5 seconds between the disks. For immediate samples, whether dry or wet, finishing and polishing were carried out immediately after curing. For 24 hour groups, after curing, samples were immersed in deionized water, and incubated in 37°C (PECO PL-455, Pooya Electronic Companic CO, Iran) for 24 hours, before finishing and polishing.

### 2.4. Assessment Procedure

3 point bending test was carried out using a universal testing machine (Zwick/Roell Z020, Ulm, Germany) for assessing flexural strength. According to ISO 4049 standard, the amount of loading force was 50 ± 16 N/min and the speed was 0.5 mm/min. The maximum amount of load that the sample was able to withstand before failure, was calculated as the flexural strength of that sample. FS was calculated from the following equation:

(1) $$\begin{array}{c} \sigma =\frac{3FL}{2bh^{2}}\text{,} \end{array}$$

where *F* is the maximum load exerted on the specimen in newton (N), *L* is the distance between supports in millimeters, *b* is the specimen width in millimeter, and *h* is the height of the specimen in millimeters. The obtained data were reported in MPa.

A microhardness test was carried out by a Vickers Machine (Zwick/Roell Indentec, Ulm, Germany). The measuring precision of the device was 0.025 microns. At first, surface of the sample was observed with 125x magnification. A smooth area was chosen and an indentation was made on it with a 200-gram load for 15 seconds. Then, the indentation was measured with 125x magnification, and microhardness was calculated. The test was carried out 3 times for each sample, with a 1.5 mm distance between indentations. The average of the three measurements was calculated as the microhardness of the sample.

Data were analyzed by Kolmogorov-Smirnov, two-way ANOVA, and Tukey HSD tests using SPSS software (IBM SPSS Statistics version 27, Armonk, NY, USA). The level of significance was *p* < 0.05 for all tests.

## 3. Results

**Figure 1:**
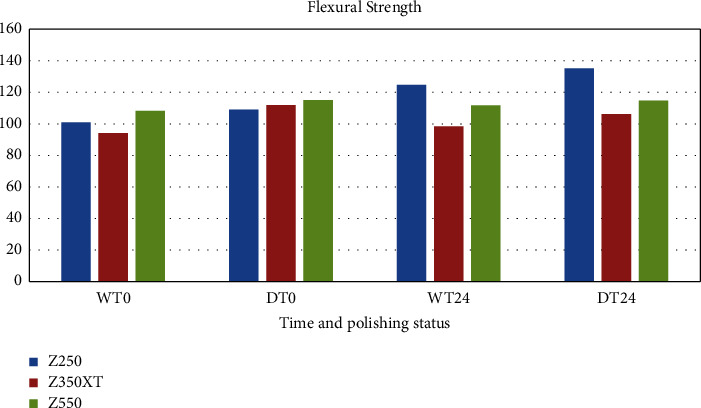
The mean values of flexural strength for three different composites at immediately (*T*_0_) and 24 hours (*T*_24_) after dry *D* and wet *W* polishing.

**Figure 2:**
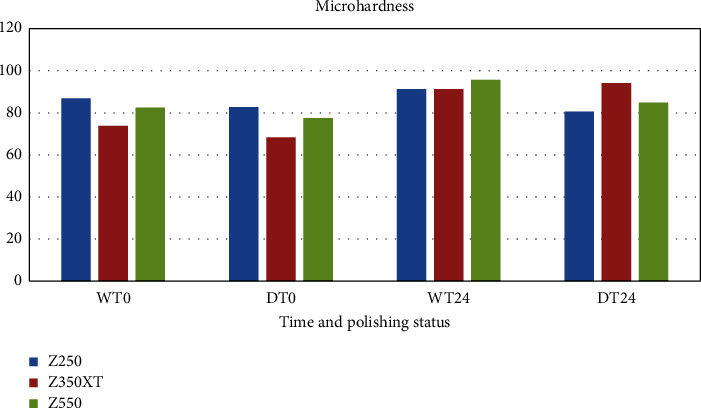
The mean values of hardness for three different composites at immediately (*T*_0_) and 24 hours (*T*_24_) after dry *D* and wet *W* polishing.

Tables 2–5 and Figures 1 and 2 show the flexural strength and hardness of composite resins subjected to different finishing and polishing protocols.

The results showed that in immediate finishing and polishing, the type of composite did not have any effect on flexural strength (*p*=0.025), but the interaction effect of finishing and polishing protocols on flexural strength values was significant (*p*=0.019) and all the dry samples showed higher flexural strength than the wet samples. In 24 hour groups, the composite type was the determining factor (*p*=0.026). The Z250 samples showed the highest flexural strength in both techniques while the Z350 XT samples showed the least.

Microhardness results showed that in immediate finishing and polishing, both the composite type and the technique were effective factors, and the microhardness of all of the wet samples was higher than the dry ones (*p*=0.008).

Tukey's test showed that in immediate finishing and polishing in both techniques, Z350 XT samples had the lowest microhardness values. In 24 hour groups, Z250 and Z550 samples, showed higher microhardness in the wet technique compared to the dry technique.

In 24 hour groups in the dry technique, the type of composite was an affective factor; Z350 XT samples had the highest microhardness values compared to Z250 and Z550 samples (*p* < 0.001), and Z550 samples were harder than Z250 (*p*=0.044).

## 4. Discussion

Surface smoothness is a property resulting from the interaction of many factors. These factors are divided into intrinsic and extrinsic factors. Intrinsic factors such as filler type, shape, size, and distribution, the type of resin matrix, the degree of final cure achieved, and the bond efficiency at the filler/matrix interface can influence the surface roughness or smoothness. Extrinsic factors are related to the type of polishing system used, such as the flexibility of the material in which the abrasives are incorporated, the hardness of the abrasives, the geometry of the instruments, and the used method [6, 10–13].

Delivering a realistic restoration which mimics natural tooth structure is one of the dentist's responsibilities. Finishing and polishing procedures can affect both the aesthetics as well as the mechanical properties of the restoration [14]. Finishing and polishing can be carried out by two methods: dry and wet. Each of these techniques has pros and cons which can affect the final properties of the restoration [15, 16].

According to the results of this study, at *T*_0,_ type of material had no effect on flexural strength, while Ramirez and Kaplan [17] and Junior et al. [18] came to the opposite conclusion. The reason may be related to the fact that they tested the flexural strength after 24 hours and after 7 days of keeping the samples in water, respectively. During this period, probably dark polymerization has taken place in the samples [9], which allows the material to show its final properties. We have carried out the test immediately after curing. At *T*_24_, we reached similar results as theirs, and our results showed a significant relationship between the type of material and flexural strength. Junior et al. [18] concluded that the filler content of composite resin materials affects their flexural strength. The factors they mentioned in this regard were weight percentage, shape, type, and sealant coverage of filler particles, and also the resin matrix composition [18]. In this study, our result showed that Z350XT as a nanofilled composite demonstrated the lowest flexural strength and highest hardness at *T*_24_ under dry conditions. Composite strength depends on the load transfer between filler and matrix. Because of forming large nano-filler aggregate in this nanofilled composite, the interfacial area between nanofiller layers and polymers are reduced and this composite cannot properly transfer the load between these 2 phases, which leads to less flexural strength [19].

Regarding the independence of flexural strength from the technique at *T*_24_, the reason may be that after dark polymerization had taken place, and cross-linking was completed, generated heat from polishing was not able to change the mechanical characteristics [20]. Also, after 24 hours, due to water absorption and the plasticizer effect of water, the strength of the samples may have altered [4].

According to the findings of the present study, at *T*_0_, wet and dry techniques had a significant effect on flexural strength, but at *T*_24_, they were irrelevant. At *T*_0_, dry samples showed higher values for flexural strength. Nasoohi et al. [21] also concluded that temperature elevation caused by dry polishing increases the amount of cross-linking and thus improves the mechanical properties.

In two of the composites, Z550 and Z250, there is actually a reduction in the surface hardness at 24 hr in the dry group compared to the wet group. These results are in accordance with the study conducted by Kaminedi et al. In his study, the hybrid resin composites (like Filtek™ Z250 and Z550) had more surface hardness under coolant compared to nanofilled composite resin (like Filtek™ Z350). This difference in the two resins may be because of the difference in the matrix and filler component of the resin. In nanofilled composites, more hardness in dry groups are expected due to the maturation of the resin matrix by the heat generated with no cooling system. However, in the hybrid composites, the particle size is larger, leaving the surface rough due to the plucking out of filler particles after wearing out of the resin matrix during polishing and the produced heat [9].

At *T*_0_, microhardness results showed a significant relationship with the type of material. Similar results to the results of the present study were observed in various studies [9, 13, 20]. In the study of Nasoohi et al. [20], both technique (wet/dry) and type of material could affect the microhardness. At *T*_24_, the technique was significantly relevant to microhardness. Nasoohi et al. [21] and Kaminedi et al. [9] have found similar results in their research. But in their studies, dry samples showed higher values, while our results showed a different outcome. This controversy may be due to the use of different polishing tools. Margio et al. [15] showed that tool characteristics can affect the mechanical properties of composites. Our polishing kit (OptiDisc, kerr, Middleton, WI, USA) may have generated less heat and thus glass transition had not taken place. Or, maybe the kit has generated a such large amount of heat that has burnt the polymer and thus caused a decreasing in the hardness. In addition, other variables can influence the hardness and flexural strength, such as curing technique and surface treatment [20, 22]. All these factors should be considered in future studies.

Another important issue is the effect of finishing and polishing time on the microleakage of composite restorations. Studies showed that the finishing and polishing time was effective on the mean microleakage in the enamel margin of composite restorations. Time of finishing and polishing had no effect on microleakage in dentin margins of restorations. Immediate finishing of the repaired restorations negatively affects the sealing at the repair interface, while 20-minute and 24-hour delayed finishing had no adverse effect on the interface sealing. The best time for finishing and polishing was 24 h after the restoration [23, 24].

The limitation of the present study involves the in-vitro methodology that can lead to a different result in clinical situations as there are other intervening factors. Also, in this study other variables such as curing technique, different polishing systems, and other mechanical factors are not considered.

Considering the limitations of this in vitro study, we can conclude that the mechanical properties of composite resins are multifactorial issues and it may not be possible to assess the effect of a single factor independently.

## 5. Conclusion

Immediate wet finishing and polishing presented lower flexural strength. Delayed dry/wet finishing and polishing significantly enhanced the hardness of the samples.

## Figures and Tables

**Table 1:** Composition, type, and manufacturer of composite resins.

Composite	Type	Matrix	Filler content %weight (wt) and %volume (vol)	Manufacturer
Z250	Microhybrid	BisGMA, UDMA, and BisEMA	82 and 60	3M, ESPE, USA
Z35oXT	Nanofilled	BisGMA, UDMA, BisEMA, TEGDMA, and PEGDMA	78.5 and 63.3	
Z550	Nanohybrid	BisGMA, UDMA,BisEMA, andTEGDMA, PEGDMA	82 and 68	

**Table 2:** Mean ± SD, minimum, median, and maximum values of flexural strength of the samples.

Materials	Groups	Mean ± SD	Minimum	Median	Maximum
Z250	*W*_T0_	100.93 ± 12.22	82.32	100.66	112.60
	*D*_T0_	109.09 ± 10.74	92.31	110.95	119.25
	*W*_T24_	124.79 ± 19.52	93.44	128.35	141.65
	*D*_T24_	135.19 ± 18.68	111.86	136.46	158.50

Z350 XT	*W*_T0_	94.20 ± 4.63	87.80	94.42	98.93
	*D*_T0_	111.96 ± 10.57	98.48	116.47	122.69
	*W*_T24_	98.38 ± 22.30	64.41	95.72	122.12
	*D*_T24_	106.17 ± 24.96	77.98	109.78	130.41

Z550	*W*_T0_	108.17 ± 13.89	90.72	107.96	128.12
	*D*_T0_	115.17 ± 16.04	88.08	120.56	129.34
	*W*_T24_	111.72 ± 18.69	87.90	119.81	129.26
	*D*_T24_	114.72 ± 22.97	76.12	118.98	134.71

*W*_0_: wet polishing at time 0, *D*_0_: dry polishing at time 0, *W*_24_: wet polishing after 24 hours, *D*_24_: dry polishing after 24 hours.

**Table 3:** Mean ± standard deviation (SD) values and result of comparison of flexural strength in three different composites in different polishing statuses (dry and wet) and times (0 and 24 hours after).

Materials	Z250	Z350XT	Z550
*W*_T0_	100.93 ± 12.22^Aa^	94.20 ± 4.63^Aa^	108.17 ± 13.89^Aa^
*D*_T0_	109.09 ± 10.74^Aa^	111.96 ± 10.57^Aa^	115.17 ± 16.04^Aa^
*W*_T24_	124.79 ± 19.52^Bb^	98.38 ± 22.30^Ab^	111.72 ± 18.69^Ab^
*D*_T24_	135.19 ± 18.68^Bb^	106.17 ± 24.96^Aa^	114.72 ± 22.97^Ab^

*W*_0_: wet polishing at time 0; *D*_0_: dry polishing at time 0; *W*_24_: wet polishing after 24 hours; *D*_24_: dry polishing after 24 hours. Means followed by different lowercase letters show statistically significant differences between them, as compared in rows. Means followed by the same uppercase letters do not show statistically significant differences between them, as compared in columns.

**Table 4:** Mean ± SD, minimum, median, and maximum values of surface hardness of the samples.

Materials	Groups	Mean ± SD	Minimum	Median	Maximum
Z250	*W*_T0_	86.86 ± 4.15	81.33	87.67	92.67
	*D*_T0_	82.73 ± 2.50	79.33	82.00	85.67
	*W*_T24_	91.26 ± 2.20	78.67	91.00	93.67
	*D*_T24_	80.60 ± 1.65	78.67	81.33	82.33

Z350 XT	*W*_T0_	73.79 ± 5.65	67.00	73.00	82.33
	*D*_T0_	68.40 ± 7.46	56.00	70.00	76.00
	*W*_T24_	91.33 ± 7.77	82.00	89.00	102.00
	*D*_T24_	94.13 ± 3.56	89.67	93.67	99.33

Z550	*W*_T0_	82.46 ± 2.61	78.33	82.67	85.00
	*D*_T0_	77.53 ± 2.31	74.67	78.33	80.33
	*W*_T24_	95.66 ± 2.86	92.33	95.67	99.67
	*D*_T24_	84.93 ± 1.84	82.67	85.00	87.67

*W*_0_: wet polishing at time 0; *D*_0_: dry polishing at time 0; *W*_24_: wet polishing after 24 hours; *D*_24_: dry polishing after 24 hours.

**Table 5:** Mean ± standard deviation (SD) values and result of comparison of hardness in three different composites in different polishing statuses (dry and wet) and times (0 and after 24 hours).

Materials	Z250	Z350XT	Z550
*W*_T0_	86.86 ± 4.15^Aa^	73.79 ± 5.65^Ab^	82.46 ± 2.61^Aa^
*D*_T0_	82.73 ± 2.50^Ba^	68.40 ± 7.46^Ab^	77.53 ± 2.31^Ba^
*W*_T24_	91.26 ± 2.20^Aa^	91.33 ± 7.77^Ba^	95.66 ± 2.86^Ca^
*D*_T24_	80.60 ± 1.65^Ba^	94.13 ± 3.56^Bb^	84.93 ± 1.84^Aa^

*W*_0_: wet polishing at time 0; *D*_0_: dry polishing at time 0; *W*_24_: wet polishing after 24 hours; *D*_24_: dry polishing after 24 hours. Means followed by different lowercase letters show statistically significant differences between them, as compared in rows. Means followed by the same uppercase letters do not show statistically significant differences between them, as compared in columns.

## Data Availability

The data used to support the study are included in the paper.
